# Costs incurred by caregivers of under-five inpatients with community-acquired pneumonia at a university hospital in south-western Ethiopia

**DOI:** 10.4102/sajid.v34i1.109

**Published:** 2019-07-29

**Authors:** Awol J. Ebrahim, Feki Naik, Fitsum S. Teni

**Affiliations:** 1 Department of Pharmacy, Institute of Health, Jimma University, Jimma, Ethiopia; 2 Department of Pharmaceutics and Social Pharmacy, School of Pharmacy, College of Health Sciences, Addis Ababa University, Addis Ababa, Ethiopia

**Keywords:** Direct medical cost, Direct non-medical cost, Ethiopia, Indirect cost, Jimma, Caregivers

## Abstract

**Background:**

Pneumonia is one of the commonest diseases among children in Ethiopia resulting in deaths and hospitalisations. The objective of the current study was to determine the cost incurred by caregivers of under-five children with community-acquired pneumonia admitted to the paediatric ward of Jimma University Specialized Hospital, south-western Ethiopia.

**Methods:**

An institution-based cross-sectional study was conducted from 01 January to 28 February 2017, through interviews with caregivers. Data on costs incurred before hospital visit, direct medical and non-medical costs, and indirect costs incurred by caregivers of the children were collected. The collected data were analysed using Statistical Package for Social Sciences version 23.

**Results:**

Among the 120 caregivers in the study, a median total cost of 304.5 Ethiopian birr (13.22 USD) was reported. This was mostly contributed by indirect costs associated with earnings lost by caregivers related to travel and stay at hospital with the children. Factors, including permanent residence, family size, hospital stay, wealth index, education and major occupation, were found to have statistically significant association with the level of cost incurred by caregivers.

**Conclusion:**

This study identified that a significant level of cost is incurred by caregivers of the children in the hospital, a majority of which was contributed by the lost earnings because of the time spent at the hospital with the children.

## Introduction

Global under-five mortality rate was 43 deaths per 1000 live births in 2015, the highest being in the World Health Organization (WHO) Africa Region.^1^ Pneumonia was among the leading causes of death among under-five children in the same year.^2^ It is caused by bacteria, virus or fungi infection manifested in the symptoms including cough, fever, chills and trouble breathing. It is more serious among children under five and the elderly, among others.^3^ In 2011, 1.3 million children died from pneumonia globally, the highest proportion of them from low-income countries.^4^ Community-acquired pneumonia (CAP) presents as an acute illness with symptoms including dyspnoea, cough and fever.^5^

Ethiopia is among the countries with highest levels of incidence of pneumonia and under-five mortality associated with it.^6^ Lower respiratory tract infections were reported to be the leading causes of under-five mortality in Ethiopia in 2013.^7^ An institution-based study in southern Ethiopia reported a prevalence of pneumonia among under-five children to be 33.5%.^8^ Another community-based study in the northwest part of Ethiopia reported a prevalence of 16.1% in the same age group.^9^ A study assessing causes of admission to a paediatric ward at a hospital in northwest Ethiopia identified CAP as the most common diagnosis.^10^ In 2010, among the 271 000 under-five children deaths in Ethiopia, about one-third was attributed to diarrhoea and pneumonia.^11^

Looking at hospitalisation associated with pneumonia, studies identified a range of findings. One study reported an incidence of hospitalisation associated with pneumonia among under five in North East of England to be 28.7%.^12^ A study from the United States also reported a nearly 10% hospitalisation rate associated with the disease in the periods 2005 and 2006.^13^ An ecological time series study in Brazil showed that between 2000 and 2011 hospital admission rate because of bacterial pneumonia among children 1 to 4 years increased from 8.50 to 14.55 in 2005 and then decreased to 11.15 in 2011.^14^

Antibiotic therapy is recommended for all children admitted with CAP. Amoxicillin is considered the first choice for oral therapy, alternative medicines being amoxicillin and clavulanate combination, cefaclor, erythromycin, azithromycin and clarithromycin. In cases where first-line treatment is not working, macrolide antibiotics can be added.^15^ As per treatment guidelines in Ethiopia, antibiotics like amoxicillin, or azithromycin are used alternatively. In case of severe pneumonia, intravenous benzyl penicillin can be used.^16^

Studies reporting on the costs associated with the management of CAP in ambulatory as well as inpatient facilities identified a range of findings in different countries. A study from France assessed the cost of managing CAP, from National Sickness Fund and patients, excluding direct non-medical costs, at a primary care. The findings came up with a mean of 118.8 euro for ambulatory care and 3522.9 euro for hospitalisation related to the disease.^17^ A study from health plan’s perspective, in the United States, which assessed cost of CAP among inpatients and outpatients, identified mean costs of 10 227 and 466 USD, respectively.^18^ A study in Florida in the United States on the cost of CAP management among adult hospitalised patients determined a mean cost of 3490 USD.^19^ Another study in the United States identified factors associated with costs of CAP treatment, with length of stay in intensive care unit connected to higher costs while adhering to treatment guidelines was associated with lower cost.^20^ Among studies which focused on a similar topic in relation to the cost of CAP for under-five children, a finding from China, which included indirect cost, reported a mean and median of 5722 and 3540 Chinese Yuan, respectively.^21^

In Ethiopia, with regard to economic aspects of pneumonia and its treatment, an extended cost-effectiveness analysis reported on the cost and associated savings of scaling up pneumococcal vaccines. An increase in the coverage of the vaccine by 10% was expected to cost 13.9 million USD, saving 2610 deaths, with a saving of 2.4 million USD in private health expenditure for households annually.^22^ Another study which assessed the out-of-pocket cost (OOP) associated with childhood pneumonia and diarrhoea, among 35 health institutions in four regions, reported mean costs of 8 and 64 USD for outpatient visits and inpatient treatments of the diseases, respectively.^23^

Apart from these studies, costs of CAP among inpatient children have not been investigated thoroughly. Documenting evidence in this area is very helpful to indicate potential areas of intervention to stakeholders. Hence, the present study aimed at assessing the costs incurred by caregivers or families of under-five children hospitalised because of CAP in the paediatric ward of Jimma University Specialized Hospital (JUSH).

## Methods

### Study setting and period

The study was conducted in the paediatric ward of JUSH, a tertiary care hospital, in Oromia Regional State, south-western Ethiopia from 01 January to 28 February 2017. It is one of the oldest public hospitals in the country located 352 km away from the capital, Addis Ababa, established in 1937. Currently, it serves approximately 15 000 inpatients, 160 000 outpatients annually. In addition, 11 000 emergency cases and 4500 deliveries a year are provided for a catchment population of about 15 million.^24^

### Study design

An institution-based cross-sectional study was conducted among caregivers of under-five children admitted to the paediatric ward of the hospital.

### Study population

The study population included all caregivers of under-five children admitted to the paediatric ward of the hospital because of CAP during data collection period of 01 January to 28 February 2017.

### Sampling

Caregivers of under-five children admitted to the paediatric ward of the hospital for CAP during the data collection period were included in the study.

### Data collection and management

Data were collected using a structured questionnaire, covering questions aimed at assessing the sociodemographic profiles, illness situations of the children under their care and spending among caregivers on direct medical services like consultations, laboratory investigations and medicines as well as indirect costs in terms of time lost while caring for the children during the illnesses. In addition, sections on direct non-medical spending on transportation, meals and lodging were included.

The data were collected by a final-year pharmacy student after a 1-day training on the contents of the instrument and interaction with potential respondents. Before the start of data collection, a pre-test of the data collection instrument was conducted among 10 respondents who were excluded from the final analysis.

Data on sociodemographic profile of the inpatients and caregivers’ OOP spending for direct medical, non-medical costs and indirect costs were collected from the perspective of caregivers. Direct medical costs were calculated by collecting data on spending on consultation, diagnostic and medicines. Direct non-medical costs included data on cost associated with transportation to the hospital, meal-related cost and cost for stay. Indirect cost was calculated based on the loss of income reported by caregivers associated with caring for the children with CAP.

### Data entry, analysis and interpretation

The data collected were entered, cleaned and analysed using SPSS version 23. Frequencies, mean and median have been employed for descriptive analysis. The economic status of the respondents and their families was categorised into five quintiles based on wealth index calculation using data on the possession of various household items and facilities. To this end, principal component analysis was performed. In the assessment of the difference in the amount of total cost among different categories of sociodemographic variables, Mann–Whitney *U* test and Kruskal–Wallis test were used as non-parametric tests. This was done because the total cost variable deviated from normal distribution. In the analyses, *p*-value less than 0.05 at 95% confidence interval was used as a cut-off point for determining statistical significance of differences.

## Ethical consideration

The study was approved by the Ethical Review Committee of Department of Pharmacy at the Institute of Health, Jimma University. Caregivers approached for the study, in the data collection process, were asked for their informed consent. Data collected from respondents did not have personal identifiers. The data collected were kept in strict confidence and used only for the purpose of the study.

## Results

### Sociodemographic profile of respondents and families

In this study, the cost associated with cases of CAP among children incurred by families visiting JUSH was investigated. In total, 120 caregivers of children were included, all of them women (100%). Among these, most were in the age group of 25–30 years (39.2%) followed by those aged 31–35 years (35.0%). More than three-quarters (78.3%) of the women were Oromo and more than 60% were Muslim in their ethnicity and religion, respectively. In terms of educational status, two-fifths (40.8%) of the participants reported not being able to read or write. Almost all of the participants (91.7%) were married and nearly two-thirds (65.0%) were housewives. As to the children, 70% were aged 2 years or younger (Table 1).

**TABLE 1:** Sociodemographic profile of respondents and family, Jimma, 2017.

Variable	Frequency(*n*)	Percentage (%)
**Age (year)**		
22–25	10	8.3
26–30	47	39.2
31–35	42	35.0
36+	21	17.5
**Sex**		
Male	0	0.0
Female	120	100.0
**Age of child (year)**		
< 1	46	38.3
1.01–2.00	38	31.7
2.01+	36	30.0
**Religion**		
Islam	73	60.8
Orthodox Christianity	34	28.3
Protestantism	13	10.8
**Ethnicity**		
Oromo	94	78.3
Amhara	6	5.0
Others	20	16.7
**Marital status**		
Married	110	91.7
Others	10	8.3
**Occupational status**		
Government employee	16	13.3
Self-employed	26	21.7
Housewife	78	65.0
**Educational status**		
Illiterate	49	40.8
Able to read and write (no formal education)	26	21.7
Completed primary school	24	20.0
Completed high school or above	21	17.5
**Family size**		
Up to five	61	50.8
Six or more	59	49.2
**Working family members**		
One	67	55.8
Two	53	44.2
**Wealth Index (quintile)**		
Lowest	24	20.0
Lower	25	20.8
Middle	23	19.2
Higher	22	18.3
Highest	26	21.7
**Permanent residence**		
Urban	31	25.8
Rural	89	74.2

Looking at the status of the family the patient and their caregivers came from, almost three-quarters (74.2%) were from outside Jimma town. In terms of generating earning, the family of 55.8% of the respondents had one member working, while the remaining respondents reported to have two members at work. The wealth index families of the participants are shown divided in to five wealth groups (quintiles) from lowest (poorest) to highest (richest) quintile (Table 1).

### Cost incurred by families of children with community-acquired pneumonia

In the study, the cost incurred by the families of the children with CAP included costs associated with actions taken before the children were brought to the hospital, cost incurred for food, transport and others during the trip to and stay at the hospital. In addition, expenditures for healthcare services in the hospital and indirect cost incurred by caregivers because of time lost because of the illness were considered.

**FIGURE 1:**
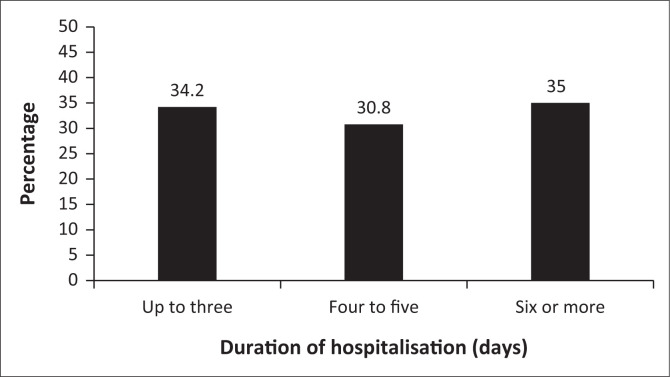
Duration of hospitalisation among patients, 2017.

Among the 120 respondents, 56.7% reported to have tried some sort of treatment to treat the child before the visit to the hospital. At the hospital, patients stayed for an average of 4.92 days (standard deviation = 2.15 days) in the ward (Figure 1).

In terms of costs associated with the action taken before the visit to hospital, the median was 0.00 (interquartile range [IQR] = 0.00–27.00) Ethiopian birr (ETB). The median cost associated to the time lost by caregivers of the children because of the disease was 100 ETB (IQR = 0.00–300.00 ETB). The direct cost components associated with the hospital visit, medical and non-medical were calculated to be a median of 80.00 and 95.00 ETB, respectively. Looking at the total cost of the illness to the families of the children, a median of 304.50 ETB (IQR = 191.25–476.25 ETB) has been incurred (Table 2).

**TABLE 2:** Cost associated with community-acquired pneumonia among children (in Ethiopian birr).

Cost type	Mean	s.d.	Median	IQR
Action taken before hospital	14.79	18.82	0.00	0.00–27.00
Indirect cost	197.23	263.62	100.00	0.00–300.00
Direct non-medical cost	94.88	55.31	95.00	62.75–120.00
Direct medical cost	93.70	50.60	80.00	62.50–109.25
**Total cost**	**400.6 ETB**	**302.45 ETB**	**304.50 ETB**	**191.25–476.25 ETB**

ETB, Ethiopian birr; IQR, interquartile range; s.d., standard deviation.

1 ETB = 0.0434 USD.

### Difference in cost incurred by sociodemographic variables

Mann–Whitney *U* test, as the median total cost was skewed from normal distribution, was done to assess the difference in the median total cost incurred related to the disease the children were suffering from. Median total cost was found to differ in a statistically significant manner by permanent residence (*p* < 0.001) with higher median cost among those who resided outside Jimma town. Median total cost showed statistically significant difference by family size (*p* < 0.001) and the number of family members (*p* = 0.006) at work (Table 3).

**TABLE 3:** Mann–Whiney *U* test of difference in the median total cost of the disease (in Ethiopian birr).

Variable	Median total cost (ETB)	*p*
**Permanent residence**		
Jimma town	205.00	< 0.001*
Out of Jimma town	343.00	
**Family size**		
Up to five	450.00	< 0.001*
Six or more	234.00	
**Number of family members a work**		
One	235.00	0.006*
Two	430.00	
**Marital status**		
Married	312.5	0.146
Other¶	203.5	

ETB, Ethiopian birr.

* *p* < 0.05

¶ , not married, separated or divorced.

Kruskal–Wallis test, non-parametric test because of non-normal distribution of the total cost, was conducted to assess the significance of difference among sociodemographic groups. Based on this, the median total cost incurred by the respondents was found to differ in a statistically significant manner among wealth index (*p* = 0.024), educational status (*p* = 0.009), hospital stay and type of major occupation (*p* < 0.001) (Table 4).

**TABLE 4:** Kruskal–Wallis test of difference in the median total cost.

Variable	Median total cost (ETB)	*p*
**Age of respondent (year)**		
22–25	417.00	0.067
26–30	417.00	
31–35	259.00	
> 35	195.00	
**Age of child (year)**		
Up to 1	308.50	0.712
1.01–2.00	310.00	
2.01 +	300.00	
**Hospital duration**		
Up to three	191.00	< 0.001*
Four to five	280.00	
Six or more	396.00	
**Wealth index**		
Lowest	255.00	0.024*
Lower	293.00	
Middle	213.00	
Higher	497.50	
Highest	404.50	
**Educational status**		
Illiterate (can’t read and write)	245.00	0.009*
Able to read and write	512.50	
Primary school	304.50	
High school of above	442.50	
**Major occupation**		
Government employee	246.00	< 0.001*
Self-employed	556.50	
Housewife	259.00	

ETB, Ethiopian birr.

* *p* < 0.05

## Discussion

This study assessed the cost incurred by families of children with CAP covering costs before coming to hospital, at hospital, as well as indirect costs. The caregivers who brought the children with pneumonia to the hospital were all women. This could be explained considering that they are mostly the parents of the children; and generally women in the area most commonly take care of and are closer to their children. In addition, the fact that most of them were housewives who were stay-at-home mothers could explain why all of them were women.

Looking at the median total cost associated with the illness experienced by the children, it was identified to be 304.5 ETB which was equivalent to 13.22 USD (1 ETB = 0.0434 USD).^25^ In 2015 and 2016, the gross domestic product (GDP) per capita of Ethiopia was reported to be 794 USD.^26^ The level of average household consumption expenditure is reported as a proxy for household income in Ethiopia. During 2015 and 2016, among households in the lowest quintile, a maximum of about 5400 ETB was reported. The average household consumption expenditure reported among those in the highest quintile was reported to be more than 15 000 ETB.^27^ According to a study done in the rural areas of Jimma Zone, the administrative area where JUSH and the surrounding areas are located, the mean total annual income was 22 214.2 (± 29 930.7).^28^ These findings indicate that the households in the study area are largely impoverished, indicating that the cost incurred by the caregivers in the present study accounts for a significant proportion of the household incomes.

The major contributor to the median total cost was indirect cost followed by direct non-medical cost. This shows that the cost associated with the disease is far reaching as it concerned time loss associated with cost and non-medical costs. The major contribution by indirect cost and direct non-medical costs could partly be associated with the fact that nearly three-quarters of the children and their caregivers came from places outside Jimma town. This contributes to higher direct costs as well as longer time lost from routine work leading to higher indirect cost.

A study done in Fiji on outpatient cost of treating pneumonia among children under five at a hospital reported that the household component of the cost was found to be an average of 10.54 USD. This was comparable but somewhat lower compared to the present study.^29^ Another study in India which assessed household cost associated with hospitalisation of infants between 2 and 36 months of age reported much higher cost in both secondary (41.35 USD) and tertiary hospital (134.62 USD). The highest contribution for the cost in the cited study was attributed to medicine costs unlike the case of the present study.^30^ Another finding from Ethiopia which assessed OOP payments associated with severe pneumonia reported a higher mean cost, 64 USD. The high cost could be associated with the consideration of severe pneumonia. In addition, the compared study included private health institutions where high medical costs are incurred.^23^

Looking at how the median total cost differed among various independent variables, it differed by the duration of hospital stay in a statistically significant manner, with higher costs incurred with longer duration. This can be related to the fact that both direct expenses and indirect costs because of the disease increase as the stay in hospital increases. In addition, permanent residence showed a statistically significant difference in terms of cost between those who lived in Jimma and outside, with higher cost incurred among those who resided outside the town. This can be associated with the higher cost incurred when travelling longer distance from outside the city to JUSH. A similar finding was reported by a study in Uganda which reported people from rural areas incurred higher cost compared to those in the urban areas because of longer travel time.^31^

Median total cost also significantly differed between those with up to five family members and those with six or more. Higher median cost was observed among respondents with up to five members. This could partly be associated with better spending power with lower number of family members. In terms of the number of productive family members earning incomes, the median total cost was higher among those with two working members compared to families with only one income earning member. The higher possibility of making more money providing higher disposable income could be among the reasons for the higher median cost associated with families with higher number of income earning members.

The median cost incurred by families associated with the illness of the children was also different in a statistically significant manner by wealth index quintile. Although no consistent pattern was observed, the median total cost was generally higher among higher quintile wealth index. This pattern could show the difference in spending power among families in different economic situations.

Educational status also showed a statistically significant difference in median total cost among respondents or families. Generally, higher education groups exhibited higher cost which could be associated with income related to higher educational status. A similarly significant association was found among different occupational groups. Generally, higher cost was observed among self-employed and government employees. This could be associated with the potential of making more money among these groups compared to housewives who depend on another person for financial assistance. Hence, spending behaviour on cost drivers in this study, indirect cost and direct non-medical cost, is likely to be higher among the former occupational groups.

## Limitation

The cost assessed through this study represents those limited to under-five inpatients and does not apply to other patients with pneumonia. The short period of data collection prevented the study from capturing possible seasonal variations in the costs incurred by caregivers.

## Conclusion

The study has been able to find out that a significant level of cost is incurred by caregivers of under-five children admitted to the hospital because of CAP. Patients living outside the town were exposed to higher costs. Higher family size, hospital stay, higher economic status and educational level were associated with increased cost. Efforts to manage cases in facilities nearer to patients’ residence could contribute to reducing costs incurred by caregivers.
